# Prevalence of Myopic Maculopathy Among Adults in a Russian Population

**DOI:** 10.1001/jamanetworkopen.2020.0567

**Published:** 2020-03-06

**Authors:** Mukharram M. Bikbov, Timur R. Gilmanshin, Gyulli M. Kazakbaeva, Rinat M. Zainullin, Ellina M. Rakhimova, Iulia A. Rusakova, Natalia I. Bolshakova, Kamila R. Safiullina, Artur F. Zaynetdinov, Ainur A. Zinatullin, Ildar F. Nuriev, Timur A. Khalimov, Songhomitra Panda-Jonas, Inga I. Arslangareeva, Guzel M. Bikbova, Dilya F. Yakupova, Yulia V. Uzianbaeva, Jost B. Jonas

**Affiliations:** 1 Ufa Eye Research Institute, Ufa, Bashkortostan, Russia; 2 Department of Ophthalmology, Medical Faculty Mannheim of the Ruprecht-Karls-University of Heidelberg, Mannheim, Germany

## Abstract

**Importance:**

Although myopic maculopathy has become a major cause of vision impairment worldwide, few data from Russia and Central Asia on the prevalence of myopic maculopathy have been available.

**Objective:**

To assess the prevalence of myopic maculopathy and its associations with ocular and systemic parameters in a population in Russia.

**Design, Setting, and Participants:**

The Ural Eye and Medical Study, a population-based case-control study, was conducted in rural and urban areas in Bashkortostan, Russia, from October 26, 2015, to July 4, 2017. Data analysis was performed from September 13 to September 15, 2019. The Ural Eye and Medical Study included 5899 of 7328 eligible individuals (80.5%) aged 40 years or older.

**Exposures:**

A detailed ocular and systemic examination included fundus photography and optic coherence tomography for the assessment of myopic maculopathy.

**Main Outcomes and Measures:**

Prevalence of myopic maculopathy.

**Results:**

The present investigation included 5794 of the 5899 eligible individuals (98.2%; 3277 [56.6%] women; mean [SD] age, 58.9 [10.7] years) with available information about myopic maculopathy. Mean (SD) axial length was 23.3 (1.1) mm (range, 19.78-32.87 mm). Prevalence of any myopic maculopathy was 1.3% (95% CI, 1.0%-1.6%); myopic maculopathy stage 2, 0.8% (95% CI, 0.6%-10.0%); stage 3, 0.2% (95% CI, 0.1%-0.4%); and stage 4, 0.2% (95% CI, 0.1%-0.4%). The prevalence of moderate to severe vision impairment and blindness was 29.8% (14 of 47 participants; 95% CI, 16.2%-43.3%) in stage 2 myopic maculopathy, 57.1% (8 of 14 participants; 95% CI, 27.5%-86.8%) in stage 3, and 100% (13 of 13 participants; 95% CI, 100%-100%) in stage 4. In multivariable analysis, a higher myopic maculopathy prevalence was associated with longer axial length (odds ratio [OR], 4.54; 95% CI, 3.48-5.92; *P* < .001), older age (OR, 1.04; 95% CI, 1.01-1.07; *P* = .03), and thinner peripapillary retinal nerve fiber layer thickness (OR, 0.96; 95% CI, 0.95-0.98; *P* < .001). After exclusion of glaucomatous eyes, the association between myopic maculopathy prevalence and thinner retinal nerve fiber layer remained significant (OR, 0.96; 95% CI, 0.95-0.98; *P* < .001). Myopic maculopathy prevalence was not significantly associated with sex; region of habitation; level of education; ethnicity; prevalence of arterial hypertension, chronic obstructive pulmonary disease, chronic kidney disease, diabetes, and inflammatory liver disease; hearing loss; depression score; or anxiety score.

**Conclusions and Relevance:**

In this ethnically mixed population from Russia, myopic maculopathy prevalence was mainly associated with elongated axial length and thinner peripapillary retinal nerve fiber layer, but was not associated with any major internal medical disease, level of education, ethnicity, or sex. Higher myopic maculopathy stage was associated with vision impairment and blindness. In addition to a known association between high axial myopia and glaucoma, myopic maculopathy may be associated with nonglaucomatous optic neuropathy.

## Key Points

**Question:**

What is the prevalence of myopic maculopathy in a Russian population?

**Findings:**

In this case-control study of 5794 individuals, the prevalence of any myopic maculopathy was 1.3%; stage 2, 0.8%; stage 3, 0.2%; and stage 4, 0.2%. A higher prevalence of myopic maculopathy was associated with longer axial length, older age, and thinner peripapillary retinal nerve fiber layer thickness, but not with any major internal medical disease, level of education, ethnicity, or sex.

**Meaning:**

In this population from Russia, myopic maculopathy prevalence was independent of educational level and major internal medical diseases, but it may be associated with nonglaucomatous optic neuropathy.

## Teaser

This case-control study examines the prevalence of myopic maculopathy among individuals aged 40 years or older in rural and urban areas in Russia.

## Introduction

Parallel to the increase in urbanization and associated changes in lifestyle, the prevalence of axial myopia has increased in the past 3 decades, in particular in East and Southeast Asia.^1^ Because myopia, mainly high myopia, can lead to marked loss in vision due to the sequelae of myopic maculopathy and high myopia–associated optic neuropathy, myopic maculopathy has become one of the most common causes of irreversible vision loss.^2,3,4,5,6,7^ Despite the importance of myopic maculopathy in public health, to our knowledge, no information has been available thus far about the prevalence of myopic maculopathy in Russia, Eastern Europe, and Central Asia, although Russia is by area the world´s largest and by population one of the most populous countries.^8^ It has also remained unclear which systemic factors and major diseases are associated with a higher prevalence of myopic maculopathy. We therefore conducted this population-based study to assess the frequency of myopic maculopathy in an ethnically mixed population in Russia and explore associations between myopic maculopathy and other ocular and systemic parameters.

## Methods

We conducted the Ural Eye and Medical Study, designed as a population-based, case-control investigation, in the Russian republic of Bashkortostan at the southwestern end of the Ural Mountains from October 26, 2015, to July 4, 2017.^9,10,11^ The inclusion criteria were age 40 years or older and living in 1 of the 2 study regions: the urban region of Kirovskii in the city of Ufa, the capital of the republic, or a rural region in the Karmaskalinsky District, which is 65 km from Ufa. Of 7328 eligible individuals, 5899 individuals (80.5%) with a mean (SD) age of 59.0 (10.7) years (range, 40-94 years) participated in the survey. Comparing the composition of the study population with data from the Russian census from 2010 did not reveal major differences in the sex and age distribution.^12^

The ethics committee of the Academic Council of the Ufa Eye Research Institute confirmed that the study design followed the tenets of the Declaration of Helsinki,^13^ and all participants gave written informed consent. Participants did not receive financial compensation.

All study participants underwent a series of examinations. Trained social workers conducted an interview including more than 250 standardized questions on the socioeconomic background, physical activity, and other parameters (eTable 1 in the Supplement). To examine potential associations between the myopic maculopathy prevalence and ethnic background, the study participants self-reported their ethnic background. The examinations included anthropometry, blood pressure measurement, handgrip dynamometry, spirometry, and biochemical analysis of blood samples taken under fasting conditions. We applied the Guidelines for Accurate and Transparent Health Estimates Reporting (GATHER) statement guidelines for collecting data in cross-sectional studies.^14^ The list of ophthalmologic examinations included, among other techniques, digital photography of the optic disc and macula (60°; Visucam 500; Carl Zeiss Meditec AG) and spectral-domain optical coherence tomography of the macula and optic nerve head (RS-3000; Nidek Co Ltd). Myopic maculopathy was graded as suggested by the Meta-analysis for Pathologic Myopia Study Group.^4^ Recent publications described the study design in detail.^9,10,11^

### Statistical Analysis

The inclusion criterion for the present study was the availability of fundus photographs for assessment of myopic maculopathy. We assessed the mean prevalence of myopic maculopathy (expressed as mean and 95% CI) and associations between myopic maculopathy prevalence and other ocular and systemic parameters, first in a univariate analysis, followed by a multivariable analysis. The latter analysis included myopic maculopathy prevalence as a dependent variable and, as independent parameters, all variables that were associated (*P* ≤ .10) with the myopic maculopathy prevalence in the univariate analyses. We then excluded parameters from the list of independent variables owing to collinearity with other independent variables or if they were no longer significantly associated with the prevalence of myopic maculopathy. We calculated odds ratios (ORs) and their 95% CIs. Data analysis was conducted from September 13 to September 15, 2019. All *P* values were 2-sided and considered statistically significant when the values were less than .05. The data were statistically analyzed using SPSS for Windows, version 25.0 (IBM-SPSS).

## Results

Of 5899 individuals primarily participating in the Ural Eye and Medical Study, the present investigation included 5794 individuals (98.2%; 3277 [56.6%] women, 2517 [43.4%] men) for whom the presence of myopic changes at the posterior fundus could be examined. The study population was composed of 1166 individuals of Russian ethnicity, 4157 individuals of non-Russian ethnicity (1049 Bashkirs, 2404 Tartars, 582 Chuvash, 21 Mari, and 101 others), and 471 individuals who did not declare their ethnic background. The mean (SD) age was 58.9 (10.7) years (median, 58 years; range, 40-94 years). The group with available fundus photographs for the assessment of myopic maculopathy compared with the group without such fundus photographs was significantly younger (mean [SD] age, 58.9 [10.7] vs 63.4 [11.3] years; *P* < .001), had a shorter axial length (mean [SD] axial length, 23.3 [1.1] vs 23.7 [1.2] mm; *P* = .001), and included a higher percentage of women (2517 men and 3277 women vs 63 men and 42 women; *P* = .001).

The mean (SD) axial length was 23.3 (1.1) mm (median, 23.2 mm; range, 19.78-32.87 mm). Defining minor myopia as an axial length of 24.0 mm or longer and less than 24.5 mm, moderate myopia as an axial length of 24.5 mm or longer and less than 26.5 mm, and high myopia as an axial length of 26.5 mm or longer, the prevalence of minor myopia was 10.4% (95% CI, 9.6%-11.2%); moderate myopia, 9.4% (95% CI, 8.7%-10.2%); and high myopia, 1.4% (95% CI, 1.1%-1.7%). Excluding pseudophakic eyes and defining minor myopia as a myopic refractive error of greater than −0.50 diopters (D) to less than −1.00 D, moderate myopia as a myopic refractive error of greater than or equal to −1.00 D to less than −8.00 D, and high myopia as a myopic refractive error of greater than or equal to −8 D, the prevalence of minor myopia was 3.8% (95% CI, 3.3%-4.3%); moderate myopia, 15.9% (95% CI, 14.9%-16.9%); and high myopia, 1.1% (95% CI, 0.8%-1.4%).

**Figure 1.**
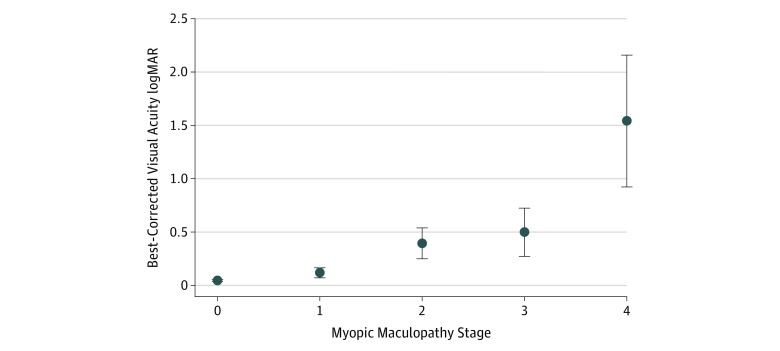
Myopic Maculopathy and Visual Acuity Association between best-corrected visual acuity and the stage of myopic maculopathy in the Ural Eye and Medical Study. Bullets indicate mean values; error bars, 95% CI. Snellen equivalents: 0.50 logMAR, 20/63, 1.00 logMAR, 20/200; 1.50 logMAR, 6/200; 2.00 logMAR, 2/200; and 2.50 logMAR, 1/200.

Among the 5794 study participants, 74 (1.3%; 95% CI, 1.0%-1.6%) individuals had any myopic maculopathy, including 47 individuals (0.8%; 95% CI, 0.6%-10.0%) with stage 2, 14 individuals (0.2%; 95% CI, 0.1%-0.4%) with stage 3, and 13 individuals (0.2%; 95% CI, 0.1%-0.4%) with stage 4 (Table 1). Best-corrected visual acuity significantly worsened with increasing stages of myopic maculopathy (Figure 1). The prevalence of moderate to severe vision impairment and blindness in participants with stage 2 myopic maculopathy was 29.8% (14 of 47 participants; 95% CI, 16.2%-43.3%); stage 3, 57.1% (8 of 14 participants; 95% CI, 27.5%-86.8%); and stage 4, 100% (13 of 13 participants; 95% CI, 100%-100%).

**Table 1.** Ocular and Systemic Parameters in the Ural Eye and Medical Study Participants

Parameter	Myopic Maculopathy, No. (%)			
	None	Stage 2	Stage 3	Stage 4
No. of patients	5720	47	14	13
Age, mean (SD), y	58.9 (10.6)	61.9 (11.8)	60.1 (12.0)	66.9 (10.5)
Sex				
Men	2489 (43.5)	17 (36.2)	6 (42.9)	5 (38.5)
Women	3231 (56.5)	30 (63.8)	8 (57.1)	8 (61.5)
Region of habitation				
Rural	3329 (58.2)	23 (48.9)	8 (57.1)	5 (38.5)
Urban	2391 (41.8)	24 (51.1)	6 (42.9)	8 (61.5)
Ethnicity				
Russian	1140 (21.7)	15 (32.6)	7 (50.0)	4 (33.3)
Non-Russian	4111 (78.3)	31 (67.4)	7 (50.0)	8 (66.7)
Alcohol consumption				
Yes	1235 (21.6)	4 (8.5)	0	2 (15.4)
No	4483 (78.4)	43 (91.5)	14 (100)	11 (84.6)
History of neck pain				
Yes	1539 (29.3)	8 (17.4)	1 (7.1)	3 (25.0)
No	3712 (70.7)	38 (82.6)	13 (92.9)	9 (75.0)
History of falls				
Yes	1067 (18.7)	15 (31.9)	3 (21.4)	3 (23.1)
No	4649 (81.3)	32 (68.1)	11 (78.6)	10 (76.9)
Serum creatinine concentration, mean (SD), mg/dL	1.02 (0.28)	0.94 (0.23)	0.99 (0.21)	0.96 (0.20)
Arterial hypertension				
Yes	4832 (84.5)	44 (93.6)	13 (92.9)	13 (100)
No	886 (15.5)	3 (6.4)	1 (7.1)	0
Dynamometric hand force, mean (SD), dekaNewton	30.6 (11.7)	28.7 (11.8)	28.3 (7.7)	24.9 (12.1)
Axial length, mean (SD), mm	23.3 (1.0)	26.3 (1.8)	27.3 (2.1)	27.9 (2.3)
Refractive error, mean (SD), D^a^	0.37 (2.10)	−6.1 (5.0)	−14.9 (6.5)	−9.8 (5.7)
Cylindrical refractive error, mean (SD), D	−0.73 (0.76)	−1.61 (1.18)	1.67 (1.27)	2.04 (1.37)
Cornea refractive power, mean (SD), D	43.9 (1.6)	42.9 (3.0)	44.4 (2.4)	43.6 (1.1)
Anterior chamber depth, mean (SD), mm^a^	3.14 (0.37)	3.33 (0.33)	3.47 (0.19)	3.90 (1.00)
Cortical cataract, prevalence^a^				
Yes	719 (14.4)	5 (15.2)	0	6 (75.0)
No	4264 (85.6)	28 (84.8)	11 (100)	2 (25.0)
Fundus tessellation, macular region, mean (SD)^b^	0.59 (0.84)	2.42 (0.92)	2.67 (0.65)	1.91 (1.38)
Retinal thickness 300 μm temporal to the fovea, mean (SD), μm	256 (40)	272 (62)	257 (73)	212 (147)
Peripapillary retinal nerve fiber layer thickness, mean (SD), μm	114 (18)	85 (23)	76 (24)	63 (23)
Open-angle glaucoma, prevalence				
Yes	131 (2.4)	5 (10.9)	1 (7.7)	3 (27.3)
No	5276 (97.6)	41 (89.1)	12 (92.3)	8 (72.7)

Abbreviation: D, diopter.

SI conversion factor: To convert serum creatinine to micromoles per liter, multiply by 88.4.

^a^ With pseudophakic eyes excluded.

^b^ Fundus tessellation was differentiated between grade 0 (no tessellation) and grade 3 (marked tessellation).

Within the axial length–defined minor myopia group, the prevalence of myopic maculopathy stage 2 was 0.7% (95% CI, 0.0%-1.4%); 1 of the eyes showed stage 3 or stage 4 of myopic maculopathy. Within the axial length–defined moderate myopia group, the prevalence of myopic maculopathy stage 2 was 2.1% (95% CI, 0.9%-3.3%); stage 3, 0.9% (95% CI, 0.1%-1.8%); and stage 4, 0.8% (95% CI, 0.0%-1.5%). Within the axial length–defined high myopia group, the prevalence of myopic maculopathy stage 2 was 32.5% (95% CI, 21.8%-43.2%); stage 3, 9.1% (95% CI, 2.5%-15.7%); and stage 4, 9.1% (95% CI, 2.5%-15.7%). Within the refractive error–defined minor myopia group, none of the eyes showed stage 2+ myopic maculopathy. Within the refractive error–defined moderate myopia group, the prevalence of myopic maculopathy stage 2 was 2.7% (95% CI, 1.6%-3.7%); stage 3, 0.6% (95% CI, 0.1%-1.0%); and stage 4, 0.8% (95% CI, 0.2%-1.4%). Within the refractive error–defined high myopia group, the prevalence of myopic maculopathy stage 2 was 16.4% (95% CI, 6.8%-26.0%); stage 3, 13.1% (95% CI, 4.4%-21.8%); and stage 4, 8.2% (95% CI, 1.1%-15.3%).

**Figure 2.**
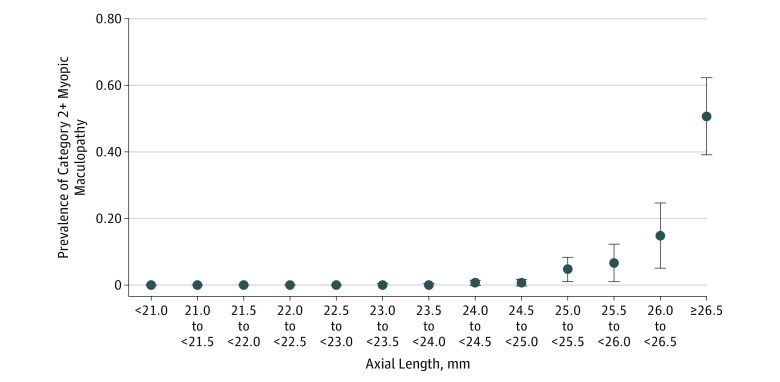
Myopic Maculopathy and Axial Length Prevalence (relative frequency) of 2+ myopic maculopathy and axial length in the Ural Eye and Medical Study. Bullets indicate mean values; error bars, 95% CI.

**Figure 3.**
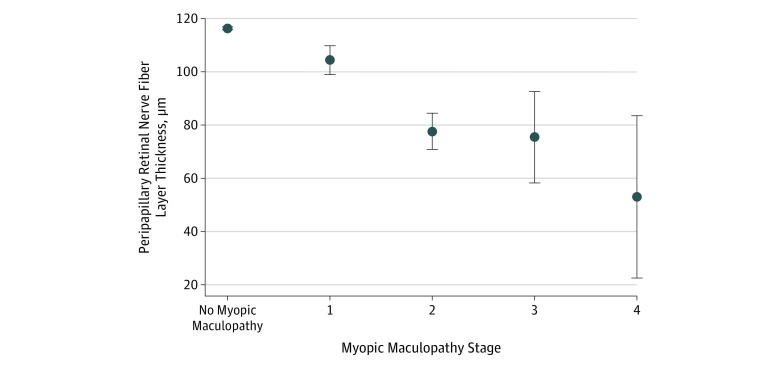
Myopic Maculopathy and Optic Nerve Association between the peripapillary retinal nerve fiber layer thickness and stage of myopic maculopathy in the Ural Eye and Medical Study. Bullets indicate mean values; error bars, 95% CI.

In univariate analysis, the prevalence of myopic maculopathy increased; systemic and ocular parameters are listed in eTable 1 and eTable 2 in the Supplement, Figure 2, and Figure 3. After we had excluded parameters owing to collinearity or a lack of statistical significance, the final model revealed that a higher prevalence of myopic maculopathy was associated with older age (OR, 1.04; 95% CI, 1.01-1.07; *P* = .03), longer axial length (OR, 4.54; 95% CI, 3.48-5.92; *P* < .001), and thinner peripapillary retinal nerve fiber layer thickness (OR, 0.96; 95% CI, 0.95-0.98; *P* < .001), in addition to a higher prevalence of falls (OR, 2.62; 95% CI, 1.24-5.51) and a lower prevalence of alcohol consumption (OR, 0.20; 95% CI, 0.06-0.63) and history of neck pain (OR, 0.27; 95% CI, 0.11-0.70) (Table 2). When we counted any axial length of less than 24 mm as 24.0 mm, the association between the prevalence of myopic maculopathy and longer axial length was significant (OR, 5.92; 95% CI, 4.27-8.21; *P* < .001). When we replaced the parameter of axial length with refractive error in the multivariable analysis and excluded all eyes that had undergone cataract surgery, we obtained similar results: a higher prevalence of myopic maculopathy was associated with higher myopic refractive error (OR, 1.56; 95% CI, 1.43-1.69; *P* < .001), but the prevalence of neck pain was no longer significantly associated (OR, 0.43; 95% CI, 0.17-1.08; *P* = .07). In the same manner, when all hyperopic refractive errors were counted as 0 D, the association between a higher prevalence of myopic maculopathy and higher myopic refractive error was significant, with a similar OR, as if the hyperopic refractive errors were counted with their original values (OR, 1.60; 95% CI, 1.47-1.76; *P* < .001). When we replaced the parameter of axial length with the ratio of axial length divided by the corneal curvature radius, the latter parameter was significantly (OR, 2.4 × 10^12^; 95% CI, 9.2 × 10^9^ to 6.2 × 10^14^; *P* < .001) associated with myopic maculopathy prevalence.

**Table 2.** Multivariable Analysis of the Prevalence of Myopic Maculopathy and With Ocular and Systemic Parameters in the Ural Eye and Medical Study

Parameter	Odds Ratio (95% CI)	*P* Value
Age, y	1.04 (1.01-1.07)	.03
Axial length, mm	4.54 (3.48-5.92)	<.001
Peripapillary retinal nerve fiber layer thickness, μm	0.96 (0.95-0.98)	<.001
Prevalence of alcohol consumption, no vs yes	0.20 (0.06-0.63)	.006
Prevalence of history of falls, no vs yes	2.62 (1.24-5.51)	.01
Prevalence of history of neck pain, no vs yes	0.27 (0.11-0.70)	.007

When peripapillary retinal nerve fiber layer thickness was dropped from the model and the prevalence of open-angle glaucoma was added, the prevalence of open-angle glaucoma was not significantly (OR, 1.06; 95% CI, 0.30-3.74; *P* = .93) associated with myopic maculopathy. When we excluded eyes with glaucomatous optic neuropathy, higher myopic maculopathy prevalence remained significantly associated with a thinner peripapillary retinal nerve fiber layer (OR, 0.96; 95% CI, 0.95-0.98; *P* < .001). When we excluded the prevalence of alcohol consumption and history of neck pain, the other parameters in the model retained the significance of their association with the prevalence of myopic maculopathy. When we added the parameters of sex; region of habitation; level of education; ethnicity; prevalence of arterial hypertension, chronic obstructive pulmonary disease, chronic kidney disease stage 3+, diabetes, and hepatitis (elevated serum concentrations of alanine aminotransferase and aspartate aminotransferase, and serum aspartate aminotransferase to alanine aminotransferase ratio); hearing loss score; depression score; and anxiety score to the model, none of these parameters was significantly associated with the prevalence of myopic maculopathy.

A higher stage of myopic maculopathy was significantly associated with longer axial length (standardized correlation coefficient β: 0.45; nonstandardized regression coefficient β: 0.15; 95% CI, 0.14-0.15; *P* < .001), thinner peripapillary retinal nerve fiber layer (standardized correlation coefficient β: −0.16; nonstandardized regression coefficient β: −0.003; 95% CI, −0.003 to 0.002; *P* < .001), and higher prevalence of a history of falls (standardized correlation coefficient β: 0.03; nonstandardized regression coefficient β: 0.02; 95% CI, 0.002-0.04; *P* = .03).

## Discussion

The ethnically mixed population of our study aged 40 years or older showed a prevalence of myopic maculopathy of 1.3% (74 of 5794 participants), with a prevalence of stage 3 myopic maculopathy of 0.2% and stage 4, 0.2%. An increasing prevalence of myopic maculopathy was associated mainly with longer axial length, in addition to older age, thinner peripapillary retinal nerve fiber layer thickness, and higher prevalence of falls, and a lower prevalence of alcohol consumption and history of neck pain. A higher myopic maculopathy stage was also associated with vision impairment and blindness.

The prevalence of myopic maculopathy of 1.3% as found in our study agrees with findings obtained in previous investigations, such as the Australian Blue Mountains Eye Study, which reported a 1.2% prevalence of myopic maculopathy.^15^ The myopic maculopathy prevalence was higher in our study population than in the population of the Central India Eye and Medical Study.^16^ The latter study was conducted in a very rural region in Central India and revealed a myopic maculopathy prevalence of 0.02% (11 of 4561 participants; 95% CI, 0.01%-0.04%). The myopic maculopathy prevalence in our study was lower than that in East Asian studies, such as in the Beijing Eye Study (prevalence of 3.1%) and in a study from Hong Kong (prevalence of 11.3%).^17,18^ The differences in myopic maculopathy prevalence between the studies are similar to differences in the prevalence of myopia and high myopia as the main risk factor for myopic maculopathy, with the lowest prevalence of high myopia in rural Central India (0.4%) and the highest prevalence in East Asian metropolitan regions (2.4%-9.1%%).^1,18,19,20,21,22,23^

The prevalence of myopic maculopathy increased by a factor of 4.54 (95% CI, 3.48-5.92) for each millimeter increase in axial length in the multivariable model in our study (Table 2; Figure 2). This finding of an association between axial length and myopic maculopathy prevalence agrees with the results of other cross-sectional investigations and with findings obtained in longitudinal studies in which longer axial length and a higher amount of axial elongation during the follow-up period was associated with a higher risk of progression of myopic maculopathy.^17,22,23,24^ In previous studies, female sex was a risk factor for a higher prevalence and a higher risk of progression of myopic maculopathy; however, in the Blue Mountains Study, Beijing Eye Study, and our investigation, the myopic maculopathy prevalence was not associated with sex.^15,16,18,23,24^ The reasons for this discrepancy among the studies have remained elusive. In our study population, parameters of the anterior ocular segment, such as keratometric readings, central corneal thickness, and anterior chamber depth and volume, were not associated with the myopic maculopathy prevalence after adjusting for axial length. This finding agrees with the results of previous histomorphometric studies in which myopic axial elongation was associated with morphologic changes in other regions, most of which were in the Southern Hemisphere.^25,26^

A higher prevalence of myopic maculopathy was correlated with a thinner peripapillary retinal nerve fiber layer in the multivariable analysis after adjusting for the presence or degree of glaucomatous optic neuropathy or after excluding eyes with glaucomatous optic nerve damage from the statistical analysis (Figure 3). Although the measurement of the peripapillary retinal nerve fiber layer can be difficult in highly myopic eyes and may lead to imprecise readings, the thinner retinal nerve fiber layer in nonglaucomatous eyes with myopic maculopathy points toward a nonglaucomatous type of optic nerve damage in highly myopic eyes with maculopathy. Although the reason for such an association has remained enigmatic, one may consider that the increase in the fovea–optic disc distance in axially elongated eyes, caused by the development and enlargement of the parapapillary gamma zone, may be associated with an elongation of the retinal ganglion cell axons. This axonal lengthening or stretching may lead to stress and eventual loss of retinal ganglion cell axons. If that assumption is valid, the risk for nonglaucomatous optic nerve damage in highly myopic eyes may increase with the width of the parapapillary gamma zone in the sector in which the retinal nerve fibers run and reach the optic nerve head.

The prevalence of myopic maculopathy was not associated with the prevalence of any major systemic diseases examined in our study. This finding agrees with previous studies and extends their results to a whole array of major internal medical diseases without association with myopic maculopathy.^1,15,16,17,18,22,23,24^ As in rural central India, the prevalence of myopic maculopathy in our study region was not associated with the level of education after adjusting for axial length as the main factor associated with the prevalence of myopic maculopathy.^16^ This finding may be of interest for the discussion of whether the educational level–associated increase in the prevalence of high axial myopia observed in the younger generations during the past 3 decades is associated with an increased risk for the eventual development of myopic maculopathy in later life. The myopic maculopathy prevalence in our study population was not associated with the prevalence of open-angle glaucoma after adjusting for axial length. This finding suggests that, other than accompanied by an elongated axial length, myopic maculopathy is not an additional risk factor for glaucoma in highly myopic eyes.

In the multivariable analysis, we excluded refractive error owing to a collinearity with axial length. One may, however, consider that axial length was significantly correlated with greater body height and with male sex, although neither body height nor sex was significantly associated with the prevalence of myopic maculopathy (eTable 1 in the Supplement). If we replaced the parameter of axial length with refractive error, we obtained a similar result for the associations between the myopic maculopathy prevalence and ocular and systemic parameters.

### Limitations and Strengths

The study has limitations. First, the quality of a population-based investigation depends on the participation rate and the representativeness of the study population. With a participation rate of 80.5% of the eligible population, a major bias in the inclusion of the participants of our study may appear unlikely. The rural and urban study regions in the southern Russian republic of Bashkortostan were typical for the whole region of southern Russia in terms of demographics, geography, and climate. With respect to the ethnic background, the percentage of Russian individuals was lower in our study region than in northwestern and central Russia. To address this limitation, we examined the prevalence of myopic maculopathy in participants of differing ethnic backgrounds in the multivariable analysis and found that the prevalence was not associated with the ethnic background. Second, the group of individuals with available fundus photographs for the assessment of myopic maculopathy differed from the group without such fundus photographs in age, axial length, and sex. The younger age and shorter axial length in the individuals with fundus photographs may have led to an underestimation of the prevalence of myopic maculopathy.

Strengths of our investigation were that, to our knowledge, it was the first population-based investigation from Russia on the prevalence of myopic maculopathy and its study sample size was relatively large. In addition, many systemic parameters were assessed and included in the statistical multivariable analysis.

## Conclusions

In this population from Russia, myopic maculopathy prevalence was 1.3% (95% CI, 1.0-1.6) with elongated axial length and thinner peripapillary retinal nerve fiber layer as the predominant associated factors. Myopic maculopathy was not associated with any major internal medical disease, level of education, or sex. However, a higher myopic maculopathy stage was associated with vision impairment and blindness. In addition to a known association between high axial myopia and glaucoma, myopic maculopathy may be associated with nonglaucomatous optic neuropathy.

## Notes

Supplement. **eTable 1.** Associations (Binary Univariate Analysis) Between the Prevalence of Myopic Maculopathy and Systemic Parameters in the Ural Eye and Medical Study **eTable 2.** Associations (Binary Univariate Analysis) Between the Prevalence of Myopic Maculopathy and Ocular Parameters in the Ural Eye and Medical Study
