# Supplementary material for: Prevalence of Myopic Maculopathy Among Adults in a Russian Population
Source: JAMA Netw Open. 2020 Mar 6;3(3):e200567. doi: 10.1001/jamanetworkopen.2020.0567 (PMC7060490; doi:10.1001/jamanetworkopen.2020.0567)
Supplement: Supplement. — eTable 1. Associations (Binary Univariate Analysis) Between the Prevalence of Myopic Maculopathy and Systemic Parameters in the Ural Eye and Medical Study eTable 2. Associations (Binary Univariate Analysis) Between the Prevalence of Myopic Maculopathy and Ocular Parameters in the Ural Eye and Medical Study [file jamanetwopen-3-e200567-s001.pdf]

Supplementary Online Content

Bikbov MM, Gilmanshin TR, Kazakbaeva GM, et al. Prevalence of myopic maculopathy among adults in a Russian population. *JAMA Netw Open*. 2020;3(3):e200567. doi:10.1001/jamanetworkopen.2020.0567

eTable 1. Associations (Binary Univariate Analysis) Between the Prevalence of Myopic Maculopathy and Systemic Parameters in the Ural Eye and Medical Study

eTable 2. Associations (Binary Univariate Analysis) Between the Prevalence of Myopic Maculopathy and Ocular Parameters in the Ural Eye and Medical Study

This supplementary material has been provided by the authors to give readers additional information about their work.

.

© 2020 Bikbov MM et al. *JAMA Network Open.*

eTable 1

Associations (binary univariate analysis) between the prevalence of myopic maculopathy and systemic parameters in the Ural Eye and Medical Study.

Parameter	Interval	Odds Ratio (OR)	95% Confidence Interval of OR	*P*-Value
Age	1-year intervals	1.03	1.01, 1.05	0.004
Gender	Men / Women	1.27	0.79, 2.03	0.33
Region of habitation	Urban / Rural	0.68	0.43, 1.08	0.10
Ethnicity	Non-Russian ethnicity / Russian	2.04	1.26, 3.31	0.004
Body height	1 cm	0.99	0.96, 1.02	0.43
Body weight	kg	0.99	0.97, 1.03	0.12
Body mass index	kg/m^2	0.97	0.93, 1.02	0.27
Waist circumference	cm	0.99	0.98, 1.01	0.50
Hip circumference	cm	0.99	0.98, 1.01	0.42
Waist/hip circumference ratio	Ratio	0.98	0.08, 12.4	0.99
Socioeconomic Score	Score	0.96	0.83, 1.12	0.61
Level of education	Illiteracy / Passing 5th Grade / 8th Grade / 10th Grade / 11th Grade / Graduates / Specialized Secondary	0.97	0.82, 1.14	0.68

© 2020 Bikbov MM et al. *JAMA Network Open.*

	Education / Post Graduates			
Physical activity Score	Score	0.97	0.94, 1.01	0.12
Smoking , currently	Yes/No	0.50	0.20, 1.25	0.14
Smoking, package years	Number	0.98	0.96, 1.01	0.20
Alcohol consumption, any	Yes/No	0.32	0.14, 0.74	0.008
In a week how many days do you eat fruits?	Number of days	0.97	0.87, 1.09	0.62
In a week how many days do you eat vegetables?	Number of days	1.00	0.85, 1.18	1.00
History of cardiovascular disorders including stroke	Yes/No	1.03	0.62, 1.74	0.90
History of angina pectoris	Yes/No	1.56	0.79, 3.05	0.20
History of asthma	Yes/No	0.48	0.07, 3.45	0.46
History of arthritis	Yes/No	0.90	0.53, 1.53	0.70
History of previous bone fractures	Yes/No	1.14	0.70, 1.87	0.61
History of low back pain	Yes/No	1.00	0.63, 1.60	0.99
History of thoracic spine pain	Yes/No	0.85	0,48, 1.50	0.57
History of neck pain	Yes/No	0.48	0.26, 0.90	0.02
History of headache	Yes/No	0.75	0.47, 1.20	0.23
History of cancer	Yes/No	1.89	0.68, 5.23	0.22
History of dementia	Yes/No	0.00	0.00, --	0.99
History of diarrhea	Yes/No	0.00	0.00, --	0.99
History of iron-deficiency anemia	Yes/No	0.23	0.03, 1.68	0.15
History of low blood pressure and hospital admittance	Yes/No	1.12	0.35, 3.59	0.85

© 2020 Bikbov MM et al. *JAMA Network Open.*

History of osteoarthritis	Yes/No	1.07	0.60, 1.93	0.82
History of skin disease	Yes/No	0.50	0.12, 2.06	0.34
History of thyreopathy	Yes/No	0.90	0.41, 1.97	0.79
History of falls	Yes/No	1.73	1.04, 2.87	0.04
History of unconsciousness	Yes/No	0.97	0.42, 2.25	0.94
Age of the last menstrual bleeding	Years	1.03	0.96, 1.10	0.46
Age of last regular menstrual bleeding	Years	1.03	0.96, 1.11	0.37
History of menopause	Yes/No	1.52	0.64, 3.62	0.34
Serum concentration of:				
Alanine aminotransferase	IU/L	1.00	0.98, 1.02	0.77
Aspartate aminotransferase	IU/L	1.00	0.98, 1.02	0.96
Aspartate aminotransferase-to-Alanine aminotransferase ratio	Ratio	0.98	0,59, 1.63	0.93
Bilirubin, total	µmol/L	1.00	0.98, 1.02	0.74
High-density lipoproteins	mmol/L	0.95	0.73, 1.24	0.70
Low-density lipoproteins	mmol/L	1.04	0.86, 1.25	0.71
Cholesterol	mmol/L	1.01	0.89, 1.15	0.84
Triglycerides	mmol/L	0.98	0.72, 1.35	0.91
Rheumatoid factor	IU/mL	1.12	0.96, 1.30	0.16
Erythrocyte sedimentation rate	Mm/min	1.00	0.98, 1.02	0.68
Glucose	mmol/L	0.95	0.81, 1.11	0.53
Urea	mmol/L	1.00	0.86, 1.17	0.99
Creatinine	µmol/L	0.99	0.98, 0.99	0.03
Hemoglobin	g/L	1.00	0.98, 1.02	0.96
Erythrocyte count	10^6 cells / µL	0.93	0.51, 1.68	0.80

© 2020 Bikbov MM et al. *JAMA Network Open.*

Leukocyte count	10^9 cells / L	1.03	0.88, 1.20	0.72
Prevalence of diabetes mellitus	Yes/No	1.06	0.52, 2.13	0.88
Estimated glomerular filtration rate	30mL/min/1.73m²	1.01	0.99, 1.02	0.46
Stage of chronic kidney disease	0-5	0.98	0.95, 1.01	0.22
Anemia	Yes/No	0.68	0.25, 1.88	0.46
Blood pressure, systolic (SBP)	mm Hg	1.01	0.99, 1.02	0.35
Blood pressure, diastolic (DBP)	mm Hg	1.01	0.99, 1.03	0.56
Blood pressure, mean	mm Hg	1.01	0.99, 1.03	0.40
Arterial hypertension	Yes/No	5.23	1.28, 21.4	0.02
Arterial hypertension, stage	0-4	1.39	1.07, 1.81	0.01
Prevalence of chronic obstructive pulmonary disease	Yes/No	0.39	0.09, 1.58	0.19
Hearing loss	Hearing loss score (0-44)	1.00	0.98, 1.02	0.90
Depression Score	Depression score unit (range: -4 to +15)	0.98	,0.92, 1.04	0.44
State-Trait Anxiety Inventory	State-Trait Anxiety Inventory Score (range: -7 to 13)	0.97	0.91, 1.04	0.36
Manual dynamometry, right hand	dekaNewton	0.98	0.96, 1.001	0.07
Manual dynamometry, right hand	dekaNewton	0.98	0.97, 0.999	0.039

© 2020 Bikbov MM et al. *JAMA Network Open.*

eTable 2

Associations (binary univariate analysis) between the prevalence of myopic maculopathy and ocular parameters in the Ural Eye and Medical Study

Parameter	Interval	Odds Ratio (OR)	95% Confidence Interval of OR	*P*-Value
Refractive error, spherical equivalent	Diopters	0.66	0.62, 0.70	<0.001
Refractive error, cylindrical value	Diopters	0.56	0.49, 0.65	<0.001
Axial length	mm	4.76	3.86, 5.88	<0.001
Corneal refractive power	Diopters	0.85	0.75, 0.96	0.01
Central corneal thickness	µm	1.00	0.99, 1.004	0.44
Corneal volume	mm^3	0.97	0.92, 1.03	0.30
Anterior chamber depth	mm	4.10	3.10, 5.43	<0.001
Anterior chamber volume	µL	1.03	1.02, 1.03	<0.001
Anterior chamber angle	Degree	1.07	1.05, 1.10	<0.001
Lens thickness	mm	1.34	0.71, 2.52	0.37
Nuclear cataract degree	Grade	1.11	0.85, 1.45	0.46
Nuclear cataract, presence	Yes/No	0.95	0.54, 1.68	0.87
Cortical cataract, degree	Percentage	1.00	0.98, 1.03	0.76
Cortical cataract, presence	Yes/No	2.04	1.07, 3.90	0.03
Subcapsular cataract, degree	Percentage	0.99	0.85, 1.15	0.85
Subcapsular cataract, presence	Yes/No	3.29	0.44, 24.6	0.25
Fundus tessellation, macula region	Grade	6.06	4.44, 8.27	<0.001

© 2020 Bikbov MM et al. *JAMA Network Open.*

Fundus tessellation, peripapillary region	Grade	6.86	4.71, 9.98	<0.001
Intraocular pressure,	mmHg	1.04	0.98, 1.10	0.16
Retinal thickness (total), fovea	µm	1.00	0.99, 1.004	0.44
Retinal thickness (total), 300 µm temporal to the fovea	µm	1.01	1.00, 1.01	0.02
Retinal thickness (total), 300 µm nasal to the fovea	µm	1.00	0.99, 1.004	0.44
Retinal nerve fiber layer thickness	µm	0.94	0.92, 0.95	<0.001
Glaucoma	Yes/No	4.08	1.99, 8.33	<0.001
Glaucoma stage	0-5	1.95	1.60, 2.36	<0.001
Open-angle glaucoma	Yes/No	5.85	2.85, 12.0	<0.001
Angle-closure glaucoma	Yes/No	0.00	0.00, ---	1.00
Diabetic retinopathy	Yes/No	2.43	0.75, 7.89	0.14
Diabetic retinopathy, ETDRS grading	Scale	1.03	0.38, 2.76	0.96

© 2020 Bikbov MM et al. *JAMA Network Open.*
